# A receptor like kinase gene with expressional responsiveness on *Xanthomonas oryzae pv. oryzae* is essential for *Xa21*-mediated disease resistance

**DOI:** 10.1186/s12284-014-0034-1

**Published:** 2015-01-17

**Authors:** Haitao Hu, Jing Wang, Chan Shi, Can Yuan, Chunfang Peng, Junjie Yin, Weitao Li, Min He, Jichun Wang, Bintian Ma, Yuping Wang, Shigui Li, Xuewei Chen

**Affiliations:** Rice Research Institute, Sichuan Agricultural University at Wenjiang, Chengdu, Sichuan 611130 China; State Key Laboratory of Hybrid Rice, Sichuan Agricultural University at Wenjiang, Chengdu, 611130 China; Collaborative Innovation Center for Hybrid Rice in Yangtze River Basin at Sichuan, Chengdu, 611130 China

**Keywords:** LRR-RLK, *Xa21*, Disease resistance, *Xanthomonas oryzae pv.oryzae*

## Abstract

**Background:**

Leucine-rich repeat receptor-like kinases (LRR-RLKs) represent a large class of proteins in regulating plant development and immunity. The LRR-RLK XA21 confers resistance to the bacterial disease caused by the pathogen of *Xanthomonas oryzae pv. oryzae* (*Xoo*). Several XA21 binding proteins have been characterized, however the early events governing XA21 signaling have not been fully elucidated.

**Results:**

Here we report the identification of one LRR-RLK gene (*XIK1*) whose expression is induced rapidly upon the infection with the pathogen of *Xoo*. Expression pattern analysis reveals that *XIK1* is preferentially expressed in reproductive leaves and panicles, and that expression is associated with plant development. By using RNA interference (RNAi), we silenced the expression of *XIK1* in rice with *Xa21* and found that reduced expression of *XIK1* compromised disease resistance mediated by XA21. In addition, we found that the expression of the downstream marker genes of pathogen associated molecular pattern (PAMP) triggered immunity (PTI) in rice was compromised in *Xa21* plants silenced for *XIK1*.

**Conclusion:**

Our study reveals that the LRR-RLK gene *XIK1* is *Xoo*-responsive and positively regulates *Xa21*-mediated disease resistance.

**Electronic supplementary material:**

The online version of this article (doi:10.1186/s12284-014-0034-1) contains supplementary material, which is available to authorized users.

## Background

Receptor-Like Kinases (RLKs) represent one of the largest protein families in plants, with more than 600 members in *Arabidopsis* (Shiu and Bleecker 2001) and 1000 members in rice (Shiu et al. 2004). A typical RLK protein contains extracellular structure, and transmembrane and kinase domains (Greeff et al. 2012). Based on the variation of the N-terminal domains, RLKs are divided into more than 44 subclasses (Gish and Clark 2011). The leucine-rich repeat (LRR)-RLK subclass is the largest, with 165 members in *Arabidopsis* (Shiu and Bleecker 2001) and 292 members in rice (Shiu et al. 2004). The LRR domain is a 24 residues-containing motif rich for leucine or other hydrophobic amino acids. Each LRR-RLK contains one or more LRRs to form a pocket used for binding to various ligands (Kolade et al. 2006; Gish and Clark 2011).

Previous studies have revealed that several LRR-RLKs are involved in plant development and immunity. For examples, the *Arabidopsis* BRI1, CLAVATA1, ERECTA1 (Clark et al. 1993; Torii et al. 1996; Clark et al. 1997; Li and Chory 1997; Jinn et al. 2000), rice BRI1 (OsBRI1) (Li and Chory 1997), and *Petunia inflate* PRK1 (Mu et al. 1994), regulate plant growth and development. The *Arabidopsis* flagellin receptor FLS2, the EF-Tu receptor EFR (Gomez-Gomez and Boller 2000; Zipfel et al. 2006), and the rice protein XA21 (Song et al. 1995) mediate immunity during plant-microbe interaction.

*Xa21* encodes a protein of rice LRR-RLK XII subclass and confers resistance against diverse strains of *Xoo* (Song et al. 1995). Although the expression of *Xa21* is quite stable during rice development, XA21*-*mediated disease resistance is development-dependent (Mazzola et al. 1994). Previous studies have shown that several XA21 binding proteins (XBs) regulate XA21-mediated immunity. These XBs belong to different protein families: such as RING finger ubiquitin ligase (XB3), transcriptional factor OsWRKY62 (XB10), protein phosphatase 2C (XB15), ATPase (XB24), endoplasmic reticulum chaperone (BiP3) and PANK protein (XB25) (Chen et al. 2010; Peng et al. 2008; Park et al. 2010; Wang et al. 2006; Park et al. 2008; Jiang et al. 2013). Recently, we also reported that OsSERK2, the rice ortholog of BAK1, regulates XA21-mediated immunity in a mechanism distinct from that of BAK1 in regulation of FLS2- and EFR-mediated immunity in *Arabidposis* (Chen et al. 2014b). Even with these advances, the mechanism of XA21-mediated immunity is still largely unknown.

Here, we characterized *XIK1* (LOC_Os02g34790), which encodes a LRR-RLK protein, through analysis of the Rice Oligonucleotide array database (ROAD: http://www.ricearray.org/). We found that the expression of *XIK1* was slightly induced upon the inoculation with *Xoo* in Kitaake but more strongly in *Xa21* plants. We found that *XIK1* is ubiquitously expressed in different rice tissues and preferentially in leaves and panicles. Transgenic *Xa21* plants silenced for *XIK1* exhibit compromised disease resistance to *Xoo*. We also found that the expression of *XIK1* is increasing during rice development, which might be the reason why XA21-mediated disease resistance is developmentally dependent. In addition, we found that the expression of marker genes for pathogen associated molecular pattern (PAMP) triggered immunity (PTI) is reduced in *Xa21* plants silenced for *XIK1.* Taken together, our study reveals that the expression of *XIK1* is *Xoo*-responsive and that XIK1 positively regulates XA21-mediated immunity.

## Results

### Identification of *XIK1*

**Figure 1:**
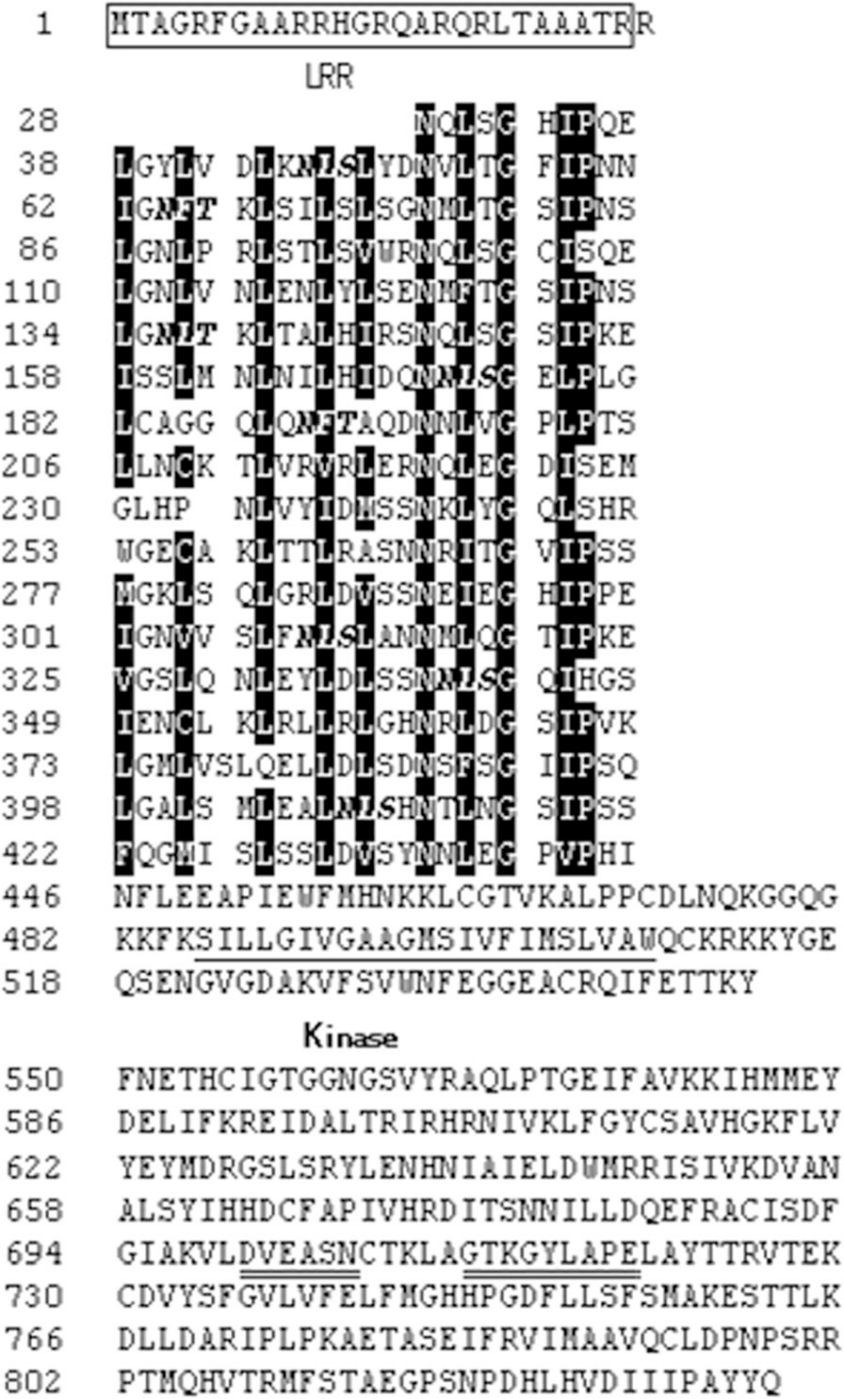
**Prediction of the amino acid sequence and protein structure of the LRR-RLK XIK1.** The sequence from amino acid residue 1 to 26 is predicted as a signal peptide. The extra-cellular region with 17 LRRs consists of the amino acid residues from 28 to 422; the amino acids characterizing the LRR motifs are marked in dark. The transmembrane region consisting of amino acid residues from 486 to 509 are underlined with single solid line. The intracellular kinase domain consisting of the amino acid residues from 550 to 802, with the conserved amino acid residues is indicated by double lines. The structure of XIK1 was predicted according to the structure of XA21 reported previously (G. L. Wang et al. 1996; Xiang et al. 2006).

LRR-RLK is a large subgroup of the rice RLK family, of which, XA21, XA3/XA26 and OsSERK2 have been reported to regulate plant immunity against *Xoo*, the causal agent of bacterial leaf blight. To determine if any other LRR-RLK is involved in the immune response against *Xoo*, we analyzed global transcriptional analysis on the expression pattern of rice LRR-RLK genes to search those with *Xoo*-responsiveness using the data available from the Rice Oligonucleotide array database (http://www.ricearray.org/). We identified eight LRR-RLK genes whose transcriptional expressions were rapidly (at 2 h post inoculation) up-regulated upon *Xoo* inoculation (Additional file 1: Figure S1). We then selected one gene, LOC_Os02g34790, for further study. The protein encoded by *LOC_Os02g34790* contains a typical signal peptide at the N-terminal, 18 repeated LRR motif and a kinase domain at the C-terminal (Figure 1). The phylogenetic tree analysis reveals that this protein belongs to LRR II subfamily (Hwang et al. 2011). We then named this gene as *Xoo-induced kinase 1* (*XIK1*).

### The expression pattern of *XIK1*

**Figure 2:**
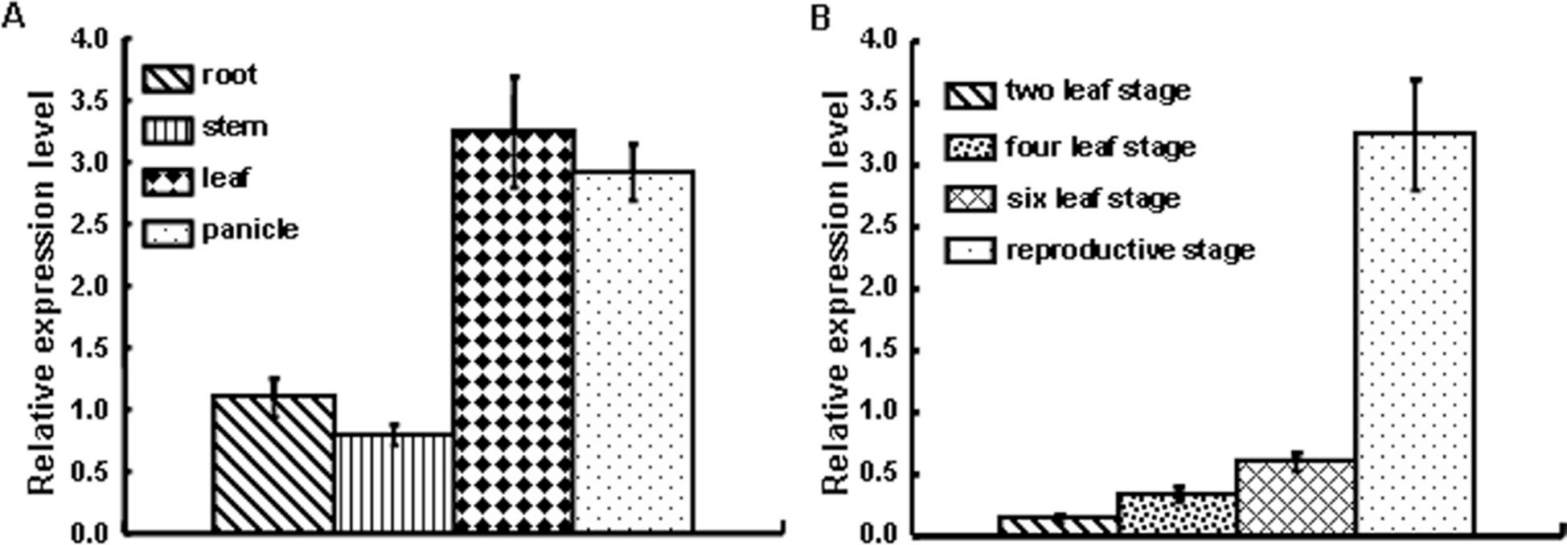
**The expressions pattern of** ***XIK1*** **. (A)**. qRT-PCR was performed on cDNA synthesized from RNA samples extracted from rice cultivar Kitaake reproductive stage tissues. Expression of *XIK1* was normalized to the expression of the *ubiquitin5* reference gene. Error bars indicating standard deviation (SD) obtained from three technical replicates. Three independent biological experiments were repeated with the similar results. **(B)**. Determination of the transcriptional expression of *XIK1* in leaves at different developmental stages as indicated. Gene expression of *XIK1* was normalized to the expression of the *ubiquitin5* reference gene. Error bars indicating SD obtained from three technical replicates. Three independent biological experiments were repeated with the similar results.

We performed quantitative RT-PCR to measure the transcription levels of *XIK1* in the rice tissues, including root, stem, leaf and panicle at the productive stage. We found that *XIK1* was ubiquitously expressed in all of the tissues with a preference in leaves and panicles (Figure 2A). We then measured the expression of *XIK1* in leaves collected from different developmental stages of rice and found that the expression of *XIK1* increases during the development of rice (Figure 2B). These results suggest that *XIK1* may function in both tissue- and development-dependent manners in rice.

### The expression of *XIK1* in *Xa21* plants is induced by *Xoo*

**Figure 3:**
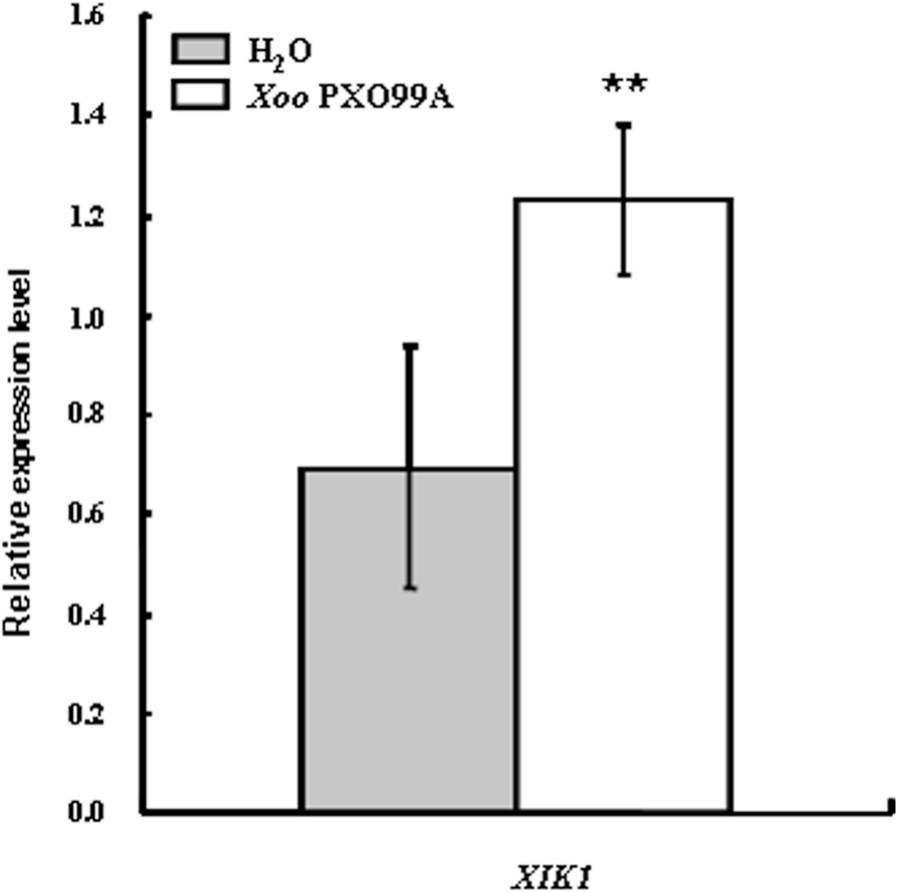
***XIK1*** **is rapidly induced by** ***Xoo*** **inoculation.** The expression of *XIK1* is induced within 2 h after *Xoo* inoculation. The ** indicate significant differences (P<0.05) as determined by a one-way ANOVA followed by post hoc Tukey HSD analysis.

As the microarray data of *XIK1* indicates that the expression of *XIK1* in Nipponbare is responsive to *Xoo,* we tested whether it could also be induced in *Xa21* plants (Peng et al. 2008). For this purpose, we collected the *Xa21* rice leaves post the inoculation with *Xoo* or H_2_O (mock-treatment) and measured the expression of *XIK1* using qRT-PCR. We found that the expression of *XIK1* was almost 1.5 folds of that obtained from mock treatment in *Xa21* resistant lines (Figure 3). This result suggests that the expression of *XIK1* in *Xa21* plants is induced upon the inoculation with *Xoo*.

### Silencing of *XIK1* compromises the XA21-mediated resistance to *Xoo*

Because the LRR-RLK XA21 functions as a pattern recognition receptor (PRR) to confer resistance to *Xoo* and *XIK1* is rapidly induced by *Xoo* inoculation in Xa21 plants, we presumed that XIK1 regulates XA21-mediated immunity. To test this hypothesis, we introduced the *XIK1Ri* construct into ProA-Xa21 homozygous rice lines to silence the expression of *XIK1* in Xa21 stable transgenic plants. ProA-Xa21 homozygous rice lines confer full resistance to the *Xoo* strain PXO99A (Chen et al. 2010). We obtained 12 independent double transgenic RNAi lines carrying both *Xa21* and *XIK1Ri* and named them *Xa21XIK1Ri* (*XXIK1Ri*) lines. Three representative lines with reduced expression levels of *XIK1* and stable expression level of the *XIK1* homolog *LOC_Os02g34750* were found (Additional file 2: Figure S2). Compared with that in the wild type *ProA-Xa21* plants, the expression of *XIK1* is reduced and is about 40%, 45% and 80% in the three *XXIK1*RNAi lines, *XXIK1Ri-1, XXIK1Ri-3* and *XXIK1Ri-8*, respectively. Expression of *LOC_Os02g34750,* with the highest identity (80.1%) of cDNA sequence to *LOC_Os02g34790,* was not altered (Additional file 2: Figure S2). We inoculated the T0 generations of these transgenic lines, and observed that the *Xa21* plants silenced for *XIK1* exhibited compromised disease resistance to the *Xoo* strain PXO99A compared with the wild type ProA-Xa21 plants (Additional file 3: Figure S3A-C). We then analyzed the disease resistance of plants from T1 generations after inoculation with PXO99A. We found that the T1 plants segregated with partial resistance in some segregants and typical full resistance in others. The double transgenic plants carrying *Xa21* and silenced for *XIK1* are partially resistant, showing typical long water-soaked lesions. The segregants carrying *Xa21* but lacking of *XIK1Ri* confer full resistance to *Xoo* as do the wild type *Xa21* plants (Additional file 3: Figure S3D). The *Xa21* plants are highly resistant and the Kitaake plants are highly susceptible to the *Xoo* strain PXO99A as expected.

**Figure 4:**
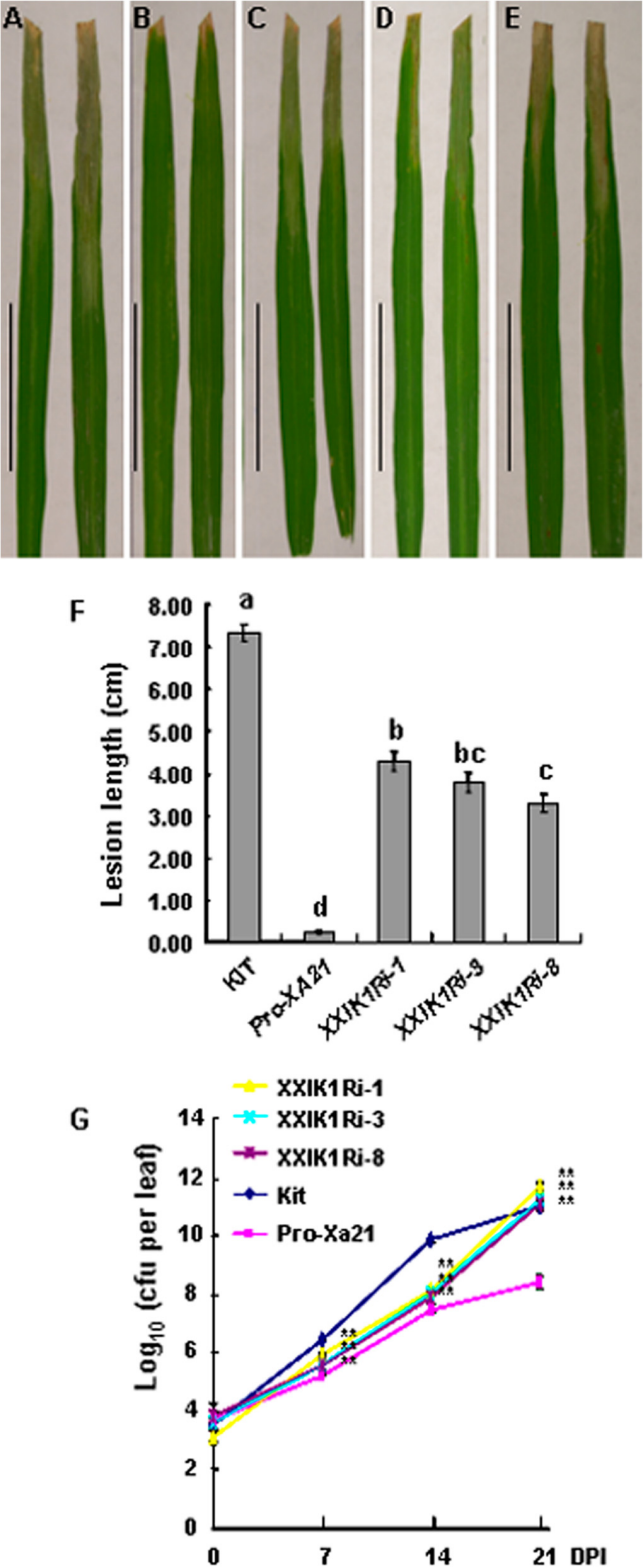
**Determination of disease resistance of ProA-Xa21 plants silenced for** ***XIK1.*** Six week-old plants were inoculated with the *Xoo* strain PXO99A. The ProA-Xa21 and Kitaake (Kit) were used as resistant and susceptible controls, respectively. Photograph depicts the representative leaves from plants at 14 DPI. The leaves from Kitaake displayed the full susceptibility to *Xoo* **(A)** whereas the leaves from ProA-Xa21 displayed full resistance to *Xoo* **(B)**. The panels **C, D,** and **E** show the representative leaves from the three transgenic lines, *XXIK1Ri-1*, *XXIK1Ri-3* and *XXIK1Ri-8,* respectively. All photographs were taken at 14 DPI. Bars= 5cm. **(F)**, Lesion lengths of *XXIK1Ri* after inoculation with *Xoo*. Lesion length was measured at 14 DPI. Graph shows average lesion length ± SD of at least 20 leaves from 10 independent plants homozygous for both *Xa21* and *XIK1Ri*. The letters indicate significant differences (P<0.05) as determined by a one-way ANOVA followed by post hoc Tukey HSD analysis. **(G)**, Bacterial cell numbers accumulated in ProA-Xa21 plants silenced for *XIK1* after inoculation with *Xoo*. Bacterial cell numbers were counted at day 0, 7, 14, 21 post inoculations. Each data point represents the average ± SD of six leaves from two independent plants. The ** indicate significant differences (P<0.05) as determined by a one-way ANOVA followed by post hoc Tukey HSD analysis. These experiments were repeated at least three times with similar results.

To characterize the effect of *XIK1* on XA21-mediated disease resistance, we performed more detailed investigations using the plants (*XXIK1Ri*) homozygous for both *Xa21* and *XXIK1Ri* derived respectively from three of the double transgenic lines. After we verified that the expression of *XIK1* was reduced whereas the expression of *Xa21* was not obviously changed in these RNAi lines (Additional file 4: Figure S4), we inoculated the plants with the *Xoo* strain PXO99AZ. At 14 days post inoculation (DPI), all of these three *XXIK1Ri* lines displayed longer lesions than the *Xa21* control (Figure 4A-E). The average lesion length of the line *XXIK1Ri-1* with the lowest expression level of *XIK1* is about 4.29 ± 0.69 cm, with the longest lesion length among these three represent *XIK1* silence lines. Meanwhile, *XXIK1Ri-8* with the highest expression of *XIK1* showed the shortest lesion length (3.29 ± 0.72) cm among these three *XIK1Ri* lines. The positive control *Xa21* plants and the negative control Kitaake plants respectively show the average lesion length of 0.26 ± 0.10 cm for the full resistance and 7.33 ± 0.10 cm for the full susceptibility as expected (Figure 4F). These results suggest that these RNAi transgenic plants exhibit partial resistance, showing typical longer water-soaked lesions than ProA-Xa21 plants but shorter than Kitaake plants.

We then quantified bacteria accumulation in plant leaves after inoculation with *Xoo* at 0, 7, 14 and 21 DPI. We found that, at 21 DPI, the bacterial numbers in *XXIK1Ri* lines were almost 100 fold (4.8×10^11^ for *XXIK1Ri-1*, 2.0×10^11^ for *XXIK1Ri-3* and 1.4×10^11^ for *XXIK1Ri-8*) greater than in ProA-Xa21 control plants (2.8×10^8^) (Figure 4G). These results are consistent with the results of disease lesion length data described above. The lesion lengths are longer and the bacterial numbers are greater when the expression level of *XIK1* is lower in the *XXIK1Ri* lines (Additional file 2: Figure S2 and Additional file 4: Figure S4), suggesting that the resistance of the transgenic lines is in accordance with the expression level of *XIK1*. Taken together, these results suggest that reduced expression of *XIK1* compromises XA21-mediated disease resistance and the LRR-LRK XIK1 plays essential roles in XA21-mediated full innate immunity in rice.

### Reduced expression of *XIK1* compromises the downstream response of PTI

**Figure 5:**
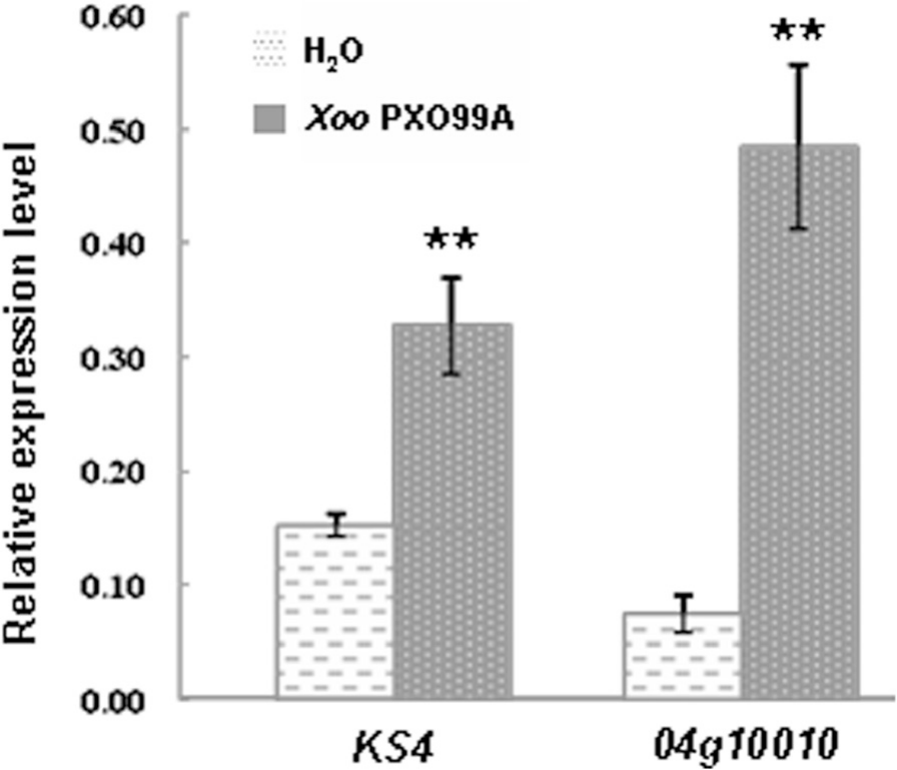
**Expression of two PTI marker genes is induced within 2 h after** ***Xoo*** **inoculation.** *OSKS4* and O*s04g10010* are rapidly induced after the infection of *Xoo* PX099A was used. Expression levels for each gene were normalized to the expression of the *ubiquitin5* reference gene. Bars depict average expression level ± SD of three technical replicates. The ** indicate significant differences (P<0.05) as determined by a one-way ANOVA followed by post hoc Tukey HSD analysis. This experiment was repeated three times with similar results.

**Figure 6:**
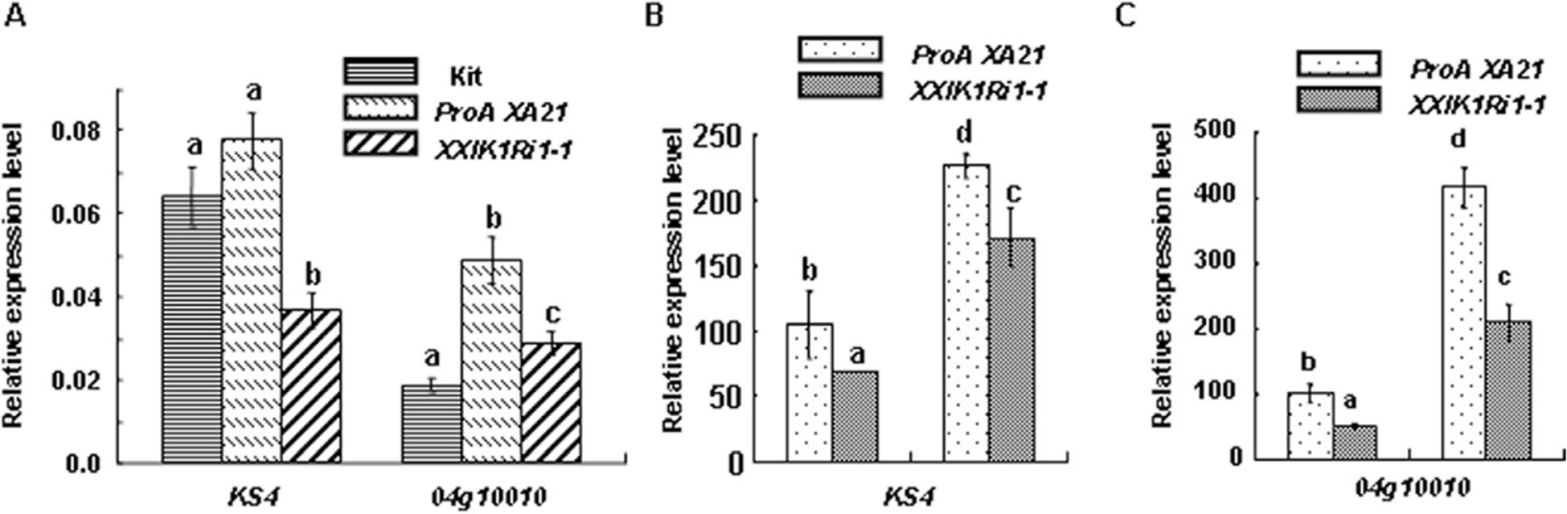
**Silencing of** ***XIK1*** **reduces the basal level of PTI marker genes in ProA-Xa21 plants** ***.*** The relative expression level of two rice PTI marker genes, *OSKS4* and *Os04g10010,* were determined by qRT-PCR using RNA extracted from rice leaves of six weeks old plants without *Xoo* inoculation **(A)** and 2 days post *Xoo* inoculation **(B, C)**. All data were normalized to the expression of the *ubiqutin5* reference gene. The results of one representative experiment were shown. Error bars indicating SD of three technical replicates. Three independent biological experiments were repeated with the similar results. The letters indicate significant differences (P<0.05) for each gene determined by a one-way ANOVA followed by post hoc Tukey HSD analysis.

Previous reports have shown that the genes, *OsKS4(LOC_Os04g10060)* and *Os04g10010(LOC_Os04g10010)*, function as marker genes involved for the downstream responses associated PTI (Park et al. 2012; Chen et al. 2014a). Thus, we asked whether these two genes in *Xa21* plants are also responsive to *Xoo*. For this purpose, we measured the expression of these two genes in rice plants post the inoculation with *Xoo* or H_2_O as a mock treatment. Compared with mock treatment, the expression levels of *OsKS4* and *Os04g10010* were induced more than two folds at 2 days post *Xoo* treatment (Figure 5). This result suggests that the XA21-mediated immune signaling was transduced downstream quickly. We then performed qRT-PCR analysis on the expression of the two genes using the leaf tissue samples collected from Kitaake, ProA-Xa21, and *XXIK1Ri-1* plants without *Xoo* inoculation. We found that the expression levels of these two genes were respectively higher in ProA-Xa21 plants than that in Kitaake plants, suggesting that rice plants carrying *Xa21* possess a higher level of basal PTI than Kitaake plants. More interestingly, we found that the expression of the two genes, *OSKS4* and *Os04g10010*, were much reduced in *XXIK1Ri-1* plants (3.6 ± 0.4 fold and 2.8 ± 0.2 fold, respectivly) compared to ProA-Xa21 plants (8.5 ± 0.1 fold and 4.0 ± 0.7 fold, respectivly) (Figure 6A). We then measured the expression of *OSKS4* and *Os04g10010* in ProA-Xa21 and *XXIK1Ri-1* plants after *Xoo* inoculation. We found that the induction of both genes were inhibited in the plants silenced for *XIK1* (Figure 6B and C). These results suggest that reduced expression of *XIK1* compromises XA21-mediated immune response both before and after *Xoo* inoculation.

## Discussion

### Expression of the LRR-RLK gene *XIK1* is responsive to *Xoo* inoculation

Although some resistant proteins have been reported to regulate rice resistance against *Xoo*, prior studies of RLK family proteins in rice were mainly focused on two RLKs: XA21 and XA3/XA26 (Chen and Ronald 2011). Phosphorylation events play critical roles for these RLKs to initiate the response triggered by PAMPs (Chen et al. 2010; Chen et al. 2014a). There are few reports showing that the expression of these RLKs is induced by pathogens and thus regulates the immune response (Century et al. 1999). Our study reveals that the RLK gene *XIK1* was induced by *Xoo* and that the encoded protein XIK1 positively regulates XA21-mediated immunity (Figures 3 and 4). Previous studies have identified some proteins that play important roles in the regulation of XA21-mediated resistance. For examples, ubiquitin E3 ligase XB3 (Wang et al. 2006) and endoplasmic reticulum chaperone BiP3 were shown to keep the XA21 protein stable (Park et al. 2014). Protein phosphatase 2C XB15 (Park et al. 2008), ATPase XB24 and rice somatic embryogenesis receptor-like kinase 2 (OsSERK2) regulate the phosphorylation of XA21(Chen et al. 2010; Chen et al. 2014a), and the WRKY transcriptional factor XB10 regulates the XA21-mediated immune response through an unknown mechanism (Peng et al. 2008). However, the expressions of these genes were not obviously changed upon the inoculation of the *Xoo* strain PXO99A. Our finding that the expression of *XIK1* was induced by *Xoo* suggests that some RLKs such as *XIK1* regulate PTI via their transcriptional expression (Figure 3). This finding provides new insights in understanding the XA21-mediated PTI signaling and the RLK-mediated innate immunities. We also found that the PTI-related marker genes were down regulated in *Xa21* plants silenced for *XIK1* (Figures 5 and 6), suggesting that the expression of the genes involved in downstream of XA21-signaling are regulated by XIK1. Additionally, since expression of *XIK1* is both tissue- and developmentally-dependent indicates that XIK1 regulates rice development and immunity tissue-specifically and developmentally (Figure 2). This result also provides the explanations why the disease resistance mediated by XA21 is developmentally-dependent.

We also performed a similar *Xoo* inoculation assay on four RNAi lines silenced for *XIK1* in Kitaake rice and found that these RNAi lines were as susceptible as Kitaake plants (data not shown). Thus, our study suggests that *XIK1* regulation of rice resistance to *Xoo* is XA21-dependent.

### XIK1 regulates early events in XA21-mediated immunity signaling

LRR-RLKs consist of the largest RLK subfamily in plants with more than 165 members in *Arabidopsis* (Shiu and Bleecker 2001) and more than 292 members in rice (Shiu et al. 2004). In rice, the well studied LRR-RLK is XA21. Investigation of the molecular mechanisms of XA21-mediated immunity have shown that many proteins are required for XA21 to function (Peng et al. 2008; Park et al. 2010; Wang et al. 2006; Park et al. 2008; Jiang et al. 2013; Chen et al. 2014a). Our study reveals that the LRR-RLK XIK1 positively regulates XA21-mediated immune signaling. As the expression of *XIK1* is induced as early as within 2 h post *Xoo* inoculation and reduced expression of *XIK1* compromises the expression of PTI-related genes, we hypothesize that XIK1 regulates the early events of XA21-mediated signaling. Because the XIK1 shares structural similarity with the LRR-RLK XA21, such as the extracellular LRR domain, a transmembrane domain that is required for the plasmembrane-localization of RLK proteins, and the intercellular active kinase domain, we suggest that XIK1 might work as a co-receptor of XA21 for PAMPs recognition and mediate the downstream signaling. As we could not detect the interaction between XIK1 and XA21 by using the intercellular domains of these two RLKs in yeast (data not shown), the extracellular domains of XIK1 and XA21 or the binding of the PAMP might be required for their interaction. To understand the precise mechanism of XIK1 in regulation of XA21-mediated immunity, it is interesting to address these questions in our future work, including whether XIK1 forms a complex with XA21, whether the extra-cellular domains of these two RLKs, or pathogen inoculation are required for their interaction, and whether OsSERK2, a recently identified RLK essential for XA21-mediated immunity is required for XIK1 to function. We also suggest that XIK1 may regulate XA3/XA26, mediated immune response to *Xoo*, as XA3/XA26 belongs to LRR XII RLK with the similar structure of XA21.

In *Arabidopsis*, the LRR-LRKs, FLS2 and EFR also mediate PTI by recognizing the PAMPs, flagellin and elongation factor (EF-tu), respectively (Gomez-Gomez and Boller 2000; Zipfel et al. 2006). Previous studies have shown that FLS2- and EFR-mediated immunities share many similar processes as XA21, such as production of reactive oxygen species (ROS), activation of mitogen activated protein kinases (MAPKs), induction of pathogenesis-related genes (PRs) (Gomez-Gomez et al. 1999; Zhang and Zhou 2010; Zipfel et al. 2006) and the requirement of the SERK proteins (i.e. SERK3/BAK1 and SERK4/BKKI for FLS2 and EFR in *Arabidopsis*, and OsSERK2 for XA21 in rice) for their early signaling (Korner et al. 2013; Yang et al. 2011; Chen et al. 2014a). We hypothesize that the RLKs, encoded by At01g35710 and At04g08850, with high identities of amino acid sequence to XIK1, might also regulate the FLS2- and EFR-mediated early signaling through a similar mechanism as that of XIK1.

LysM-RLKs, another RLK subgroup, have been shown to mediate PTI in rice and *Arabidopsis* (Miya et al. 2007; Shimizu et al. 2010; Chen and Ronald 2011). The LysM conserved motif of these receptors is reported to mediate plant-pathogen interactions by recognizing chitin molecules derived from fungal pathogens such as *Magnaporthe oryzae* (Chen and Ronald 2011). Thus, it would also be of great interest to determine whether XIK1 or other proteins with a similar structure of XIK1 regulate LysM-RLK-mediated immunity in the future work.

## Conclusion

Our study revealed that the LRR RLK gene *XIK1* is pathogen responsive and its expression is induced rapidly upon the inoculation with *Xoo*. Silencing of *XIK1* comprises XA21-mediated resistance to *Xoo* and XIK1 positively regulates XA21-mediated resistance.

## Methods

### Plant growth, *Xoo* inoculation and disease resistance determination

All of the plants were grown in rice fields (Wenjiang, Chengdu, Sichuan, China). Rice Kitaake is used for transcriptional expression pattern analysis. The silencing construct of *XIK1* (*XIK1RNAi*) was introduced into the *Xa21* (ProA-Xa21) plants with full resistance to the *Xoo* strain PXO99A (Hopkins et al. 1992) to obtaine double transgenic plants containing *Xa21* and *XIK1RNAi*. Plants were inoculated with the *Xoo* strain PXO99A. The inoculation and disease lesion length determination was performed according to the methods described previously (Song et al. 1995). Statistical analysis was performed using a one-way ANOVA followed by post hoc Tukey HSD. The leaves inoculated with *Xoo* were collected and prepared for transcriptional expression analyses as described below.

### RNA extraction and real time RT-PCR analyses

Total RNA was isolated from rice plant tissues using Invitrogen RNA isolation reaction, TRIzol (Invitrogen), following the procedures of the manual. The first strand cDNA was synthesized using the Takara reverse transcription kit (Takara). Quantitative real time PCR (qRT-PCR) was performed on a Bio-Rad CFX96 Real-Time System coupled to a C1000 Thermal Cycler (Bio-Rad). For qRT-PCR reactions, the SsoFastEva Green Super mix was used. The gene expression levels relatively to the *ubiquitin5* gene (*LOC-06g46770*) were analyzed using delta-delta ct method. All of the qRT-PCR primers are shown in Additional file 5: Table S1.

### Constructions

For *XIK1* RNAi construct, a 361 bp unique cDNA sequence (from 72 nt to 432 nt) of *XIK1Ri* amplified by primer pair *XIK1*Ri-1/-2 (shown in Additional file 5: Table S1) and was cloned into **_**pCR®8™⁄GW/TOPO® (Invitrogen) vector to create the construct *pCR8-XIK1Ri. pCR8-XIK1Ri* was then inserted into pANDA (Miki and Shimamoto 2004) through LR recombination to create the construct pANDA-*XIK1Ri*.

### Generation of transgenic rice plants

The RNAi construct for *XIK1,* pANDA-*XIK1Ri,* was introduced into *Xa21* plants through *Agrobacterium*-mediated transformation according to the method described previously (Chern et al. 2005). Because the ProA-Xa21 transgenic plants are mannose resistant, *XIK1Ri* transgenic plants in *Xa21* genetic background were selected with hygromycin in our study.

### Accession numbers

RGAP: LOC_Os02g34790, LOC_Os02g34750

## Additional files

Additional file 1: Figure S1. Identification LRR-RLKs induced by *Xoo* inoculation in 2 hours. These genes were identified from the array data (http://www.ricearray.org/) of experiment “Comparative transcriptional profiling of rice undergoing infection by *X.oryzae* pv *oryzae* or by *X.oryzae* pv.*oryzicola*”. We selected the LRR-RLKs which were specially induced by *Xoo* strain PXO99A in susceptible variety *Nipponbare*. Additional file 2: Figure S2. Identification of transgenic plants with reduced expression of *XIK1*. (A) A special fragment was chosen for *XIK1* RNAi construct. (B) The relative expression level of *XIK1* was determined by qRT-PCR using RNA extracted from rice leaves of RNAi transgenic lines as indicated and ProA-Xa21 plants. All data were normalized to the expression of the *ubiqutin5* reference gene. The average expression level from one representative biological experiment was shown. Error bars indicate SD of three technical replicates. Three independent biological experiments were repeated with the similar results obtained. Additional file 3: Figure S3. Determination of the resistance of the T0 and T1 generation of *XIK1Ri* transgenic plants to *Xoo*. Six weeks-old plants were inoculated with the *Xoo* strain PXO99A. The ProA-Xa21 and Kitaake (Kit) were used as resistant and susceptible controls, respectively. From (A) through (C), T0 Transgenic plants carrying *XIK1Ri* developed long water-soaking lesions. Photograph depicts the representative leaves from the plants at 14 DPI. (D), Lesion length was measured 14 days post inoculation with *Xoo* strain PXO99A. “*Ri(+)*” indicates T1 segregants carrying the transgene *XIK1Ri* whereas “*Ri(−)*” indicates T1 segregants lacking of *XIK1Ri*. This experiment was repeated three times with similar results. Additional file 4: Figure S4. Expression level of *Xa21* in the plants silenced for *XIK1* by qRT-PCR. All data were normalized to the expression of the *ubiqutin5* reference gene. Additional file 5: Table S1. The primers used in this study.

## Abbreviations

DPI: Days post inoculation
*XIK1*: *Xoo-induced kinase 1*
