# Supplementary figures and images for: A receptor like kinase gene with expressional responsiveness on Xanthomonas oryzae pv. oryzae is essential for Xa21-mediated disease resistance
Source: Rice (N Y). 2015 Jan 17;8:1. doi: 10.1186/s12284-014-0034-1 (PMC4883590; doi:10.1186/s12284-014-0034-1)

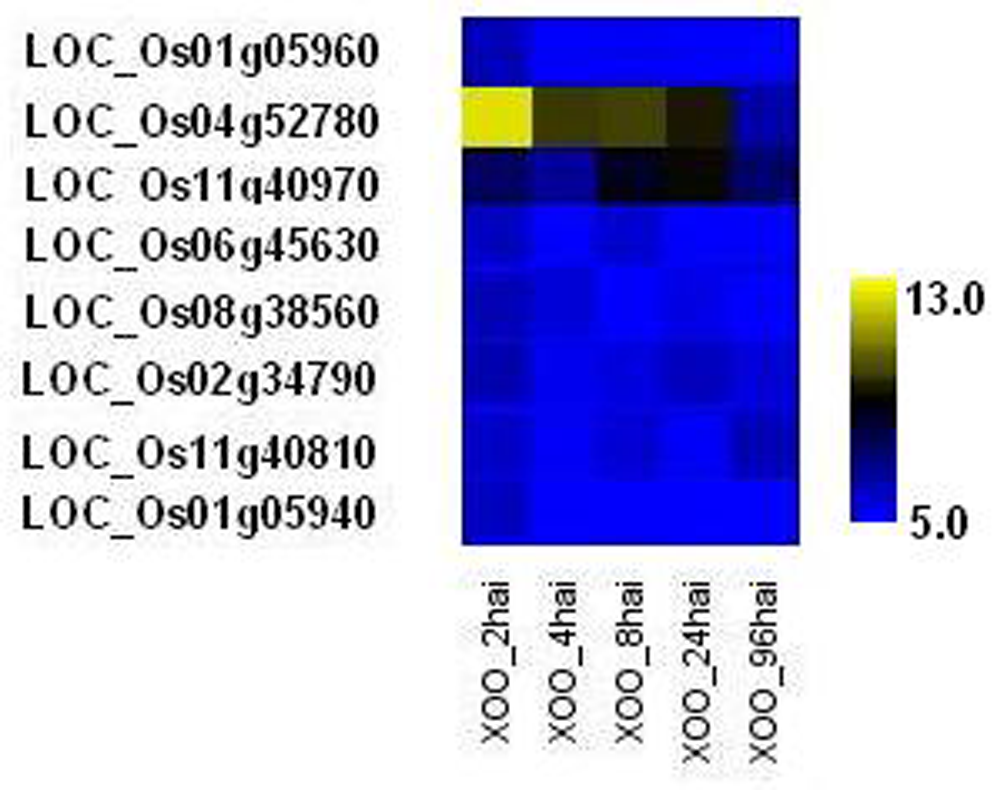

Supplement: Additional file 1: Figure S1. — Identification LRR-RLKs induced by Xoo inoculation in 2 hours. These genes were identified from the array data (http://www.ricearray.org/) of experiment “Comparative transcriptional profiling of rice undergoing infection by X.oryzae pv oryzae or by X.oryzae pv.oryzicola”. We selected the LRR-RLKs which were specially induced by Xoo strain PXO99A in susceptible variety Nipponbare. [file 12284_2014_34_MOESM1_ESM.tiff]

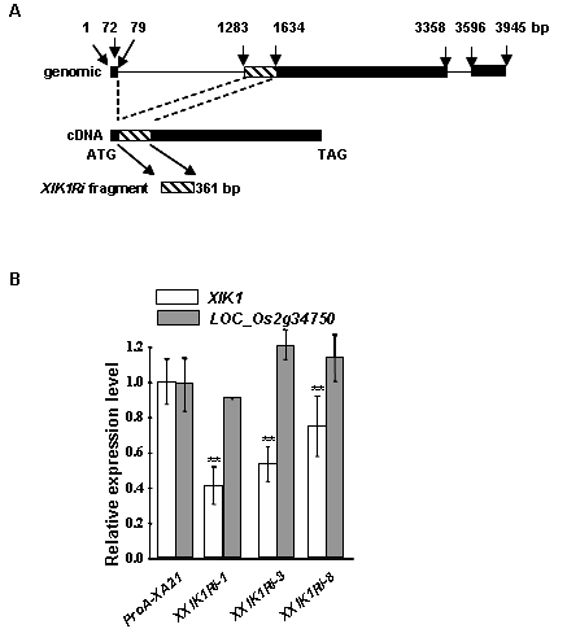

Supplement: Additional file 2: Figure S2. — Identification of transgenic plants with reduced expression of XIK1. (A) A special fragment was chosen for XIK1 RNAi construct. (B) The relative expression level of XIK1 was determined by qRT-PCR using RNA extracted from rice leaves of RNAi transgenic lines as indicated and ProA-Xa21 plants. All data were normalized to the expression of the ubiqutin5 reference gene. The average expression level from one representative biological experiment was shown. Error bars indicate SD of three technical replicates. Three independent biological experiments were repeated with the similar results obtained. [file 12284_2014_34_MOESM2_ESM.tiff]

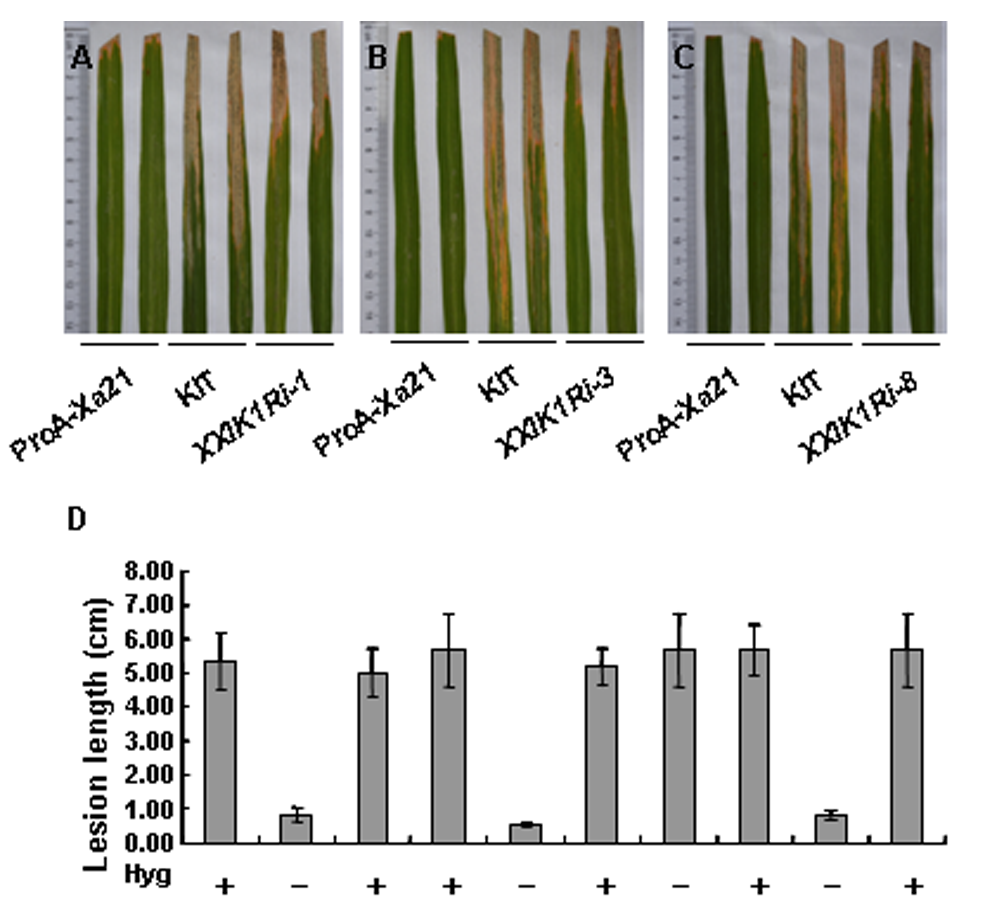

Supplement: Additional file 3: Figure S3. — Determination of the resistance of the T0 and T1 generation of XIK1Ri transgenic plants to Xoo. Six weeks-old plants were inoculated with the Xoo strain PXO99A. The ProA-Xa21 and Kitaake (Kit) were used as resistant and susceptible controls, respectively. From (A) through (C), T0 Transgenic plants carrying XIK1Ri developed long water-soaking lesions. Photograph depicts the representative leaves from the plants at 14 DPI. (D), Lesion length was measured 14 days post inoculation with Xoo strain PXO99A. “Ri(+)” indicates T1 segregants carrying the transgene XIK1Ri whereas “Ri(−)” indicates T1 segregants lacking of XIK1Ri. This experiment was repeated three times with similar results. [file 12284_2014_34_MOESM3_ESM.tiff]

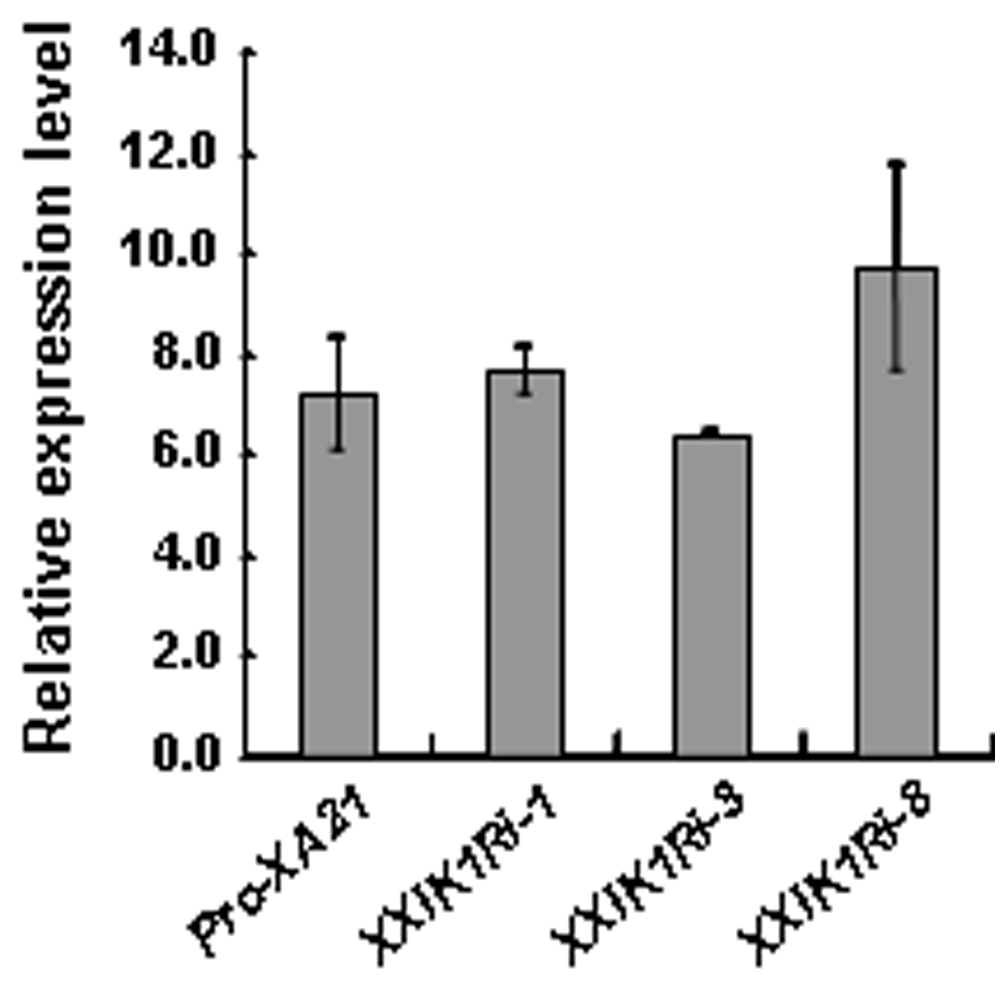

Supplement: Additional file 4: Figure S4. — Expression level of Xa21 in the plants silenced for XIK1 by qRT-PCR. All data were normalized to the expression of the ubiqutin5 reference gene. [file 12284_2014_34_MOESM4_ESM.tiff]
