# Supplementary material for: A receptor like kinase gene with expressional responsiveness on Xanthomonas oryzae pv. oryzae is essential for Xa21-mediated disease resistance
Source: Rice (N Y). 2015 Jan 17;8:1. doi: 10.1186/s12284-014-0034-1 (PMC4883590; doi:10.1186/s12284-014-0034-1)
Supplement: Additional file 5: Table S1. — The primers used in this study. [file 12284_2014_34_MOESM5_ESM.doc]

**Supplemental table S1: The primers used in this study**

| **purpose** | | **gene** | | | | **ID** | **name** | **squence (5 ' -3 ')** |
| --- | --- | --- | --- | --- | --- | --- | --- | --- |
| RT-PCR | | *XXIK1*Ri | | [*LOC_Os02g34790*](http://rice.plantbiology.msu.edu/cgi-bin/ORF_infopage.cgi?orf=LOC_Os04g10060.1) | | | F | TTGGGCCATTGCCAACAAGC |
|  | |  | | | | | R | GTGGTAAGTTTGGCGCACTC |
|  | | *OsKS4* | |  | [*LOC_Os04g10060*](http://rice.plantbiology.msu.edu/cgi-bin/ORF_infopage.cgi?orf=LOC_Os04g10060.1) | | F | TCGCATTGCGTGTGCAA |
|  | | |  | | | | R | TTGGAACTTCCGACATCGAAA |
|  | *04g10010* [*LOC_Os04g10010*](http://rice.plantbiology.msu.edu/cgi-bin/ORF_infopage.cgi?orf=LOC_Os04g10060.1) | | | | | | F | AAATGATTTGGGACCAGTCG |
|  |  | | | | | | R | GATGGAATGTCCTCGCAAAC |
|  | *OsUBQ5* | | | *LOC_Os06g46770* | | | F | CCAGTAAGTCCTCAGCCATGGA |
|  |  | | | | | | R | GGACACAATGATTAGGGATC |
| *pANDA:XIK1* | *XIK1*Ri | | | [*LOC_Os02g34790*](http://rice.plantbiology.msu.edu/cgi-bin/ORF_infopage.cgi?orf=LOC_Os04g10060.1) | | | F | GACCAGGCGAAATCAACTTT |
|  |  | | | | | | R | ATGTAAGGCAGTGAGTTTAGTCAA |
